# The greatest loss of unpleasant smells may be related to the risk of more severe PD symptoms

**DOI:** 10.3389/fneur.2024.1362763

**Published:** 2024-04-02

**Authors:** Shih-Chi Chiu, Ting-Chun Fang, Hsin-Bei Lei, Yu-Hsuan Lin, Ming-Hong Chang, Yi-Jen Guo

**Affiliations:** ^1^Department of Neurological Institute, Taichung Veterans General Hospital, Taichung, Taiwan; ^2^Department of Post-Baccalaureate Medicine, College of Medicine, National Chung Hsing University, Taichung, Taiwan; ^3^Brain and Neuroscience Research Center, College of Medicine, National Chung Hsing University, Taichung, Taiwan

**Keywords:** Parkinson’s disease, hyposmia, UPSIT, odor emotional valence, motor subtypes

## Abstract

**Background:**

Limited research has explored the relationship between the valence of olfactory dysfunction and PD clinical symptoms. This study aimed to investigate correlations between the emotional valence of olfactory impairment and different domains of PD symptoms.

**Methods:**

PD patients who fulfilled the clinically probable PD diagnostic criteria of the International Parkinson and Movement Disorder Society Clinical Diagnostic Criteria for Parkinson’s Disease were recruited from the Center for Parkinson and Movement Disorders at Taichung Veterans General Hospital between October 2016 and April 2022. Demographic data and serial clinical assessments were collected, including the traditional Chinese version of the University of Pennsylvania Smell Identification Test (UPSIT-TC) and Movement Disorder Society-sponsored revision of the Unified Parkinson’s Disease Rating Scale (MDS-UPDRS). Thirty-five odors from the UPSIT-TC were classified into neutral, pleasant or unpleasant groups. Group comparisons, correlation analyses, and linear regression analyses were conducted to examine the relationship between olfactory impairment of UPSIT-TC odors, considering emotional valence, and MDS-UPDRS subscores across various domains.

**Results:**

A total of 176 PD patients were recruited for analysis. Patients in the predominantly neutral/unpleasant odor impairment groups had higher MDS-UPDRS part III scores compared to those in the predominantly pleasant odor impairment group (pleasant vs. neutral vs. unpleasant odor impairment groups: 26.79 ± 13.59 vs. 35.33 ± 16.36 vs. 31.57 ± 12.37, *p* = 0.009). This trend was also noted in MDS-UPDRS rigidity, bradykinesia, and akinetic-rigid subscores (*p* = 0.003, *p* = 0.012, and *p* = 0.001, respectively). Correlation analysis revealed a weak but significant correlation between rigidity/akinetic-rigid subscores and misidentification numbers for neutral/unpleasant odors (all *p* < 0.05), with age, gender, LEDD, and disease duration as covariates. All significances were retained in the linear regression analysis.

**Conclusion:**

Our results emphasize the link between olfactory impairment of specific emotional valence, neutral/unpleasant odors, and PD severity, particularly with respect to akinetic-rigid symptoms. A concise olfactory test that focuses on both neutral and unpleasant odors may offer deeper insights into PD symptoms.

## 1 Introduction

Parkinson’s disease (PD) is a chronic neurodegenerative disorder characterized by several motor and non-motor symptoms, of which olfactory dysfunction is the most frequent non-motor symptom, occurring in 96% of PD patients (1) and often preceding motor symptoms (2). Olfactory dysfunction is recognized as a supportive criterion for PD and is included in the International Parkinson and Movement Disorder Society Clinical Diagnostic Criteria (3). Moreover, hyposmia has been associated with reductions in striatal dopaminergic function in PD (4).

While some studies have reported an association between hyposmia and disease severity as measured by the Movement Disorder Society Unified Parkinson’s Disease Rating Scale (MDS-UPDRS) part III score (5–7), others have found no significant correlation between the extent of hyposmia and the stage or severity of the disease (7, 8). In addition, a correlation study between olfactory function and non-motor symptoms in PD reported that hyposmia was more consistently related to executive and autonomic dysfunction (7, 9). Hedonic appreciation of pleasant odors has been reported to decrease relatively earlier than unpleasant smells in older adults compared to young adults (10), and a similar phenomenon has been reported in PD (11). The importance of the valence of olfactory dysfunction may differ in different neurodegenerative processes (11). However, there is limited research on the relationship between differences in the valence of olfactory dysfunction and the severity of both motor and non-motor symptoms in PD. Therefore, the aim of this study was to investigate the relationships between different domains of clinical symptoms and emotional valence of olfactory impairment in PD.

## 2 Materials and methods

### 2.1 Participants

We recruited PD patients from the Center for Parkinson and Movement Disorders at Taichung Veterans General Hospital (VGHTC) in this retrospectively cross-sectional study between October 2016 and April 2022. In this study, 176 PD patients (96 males and 80 females) were enrolled, with an average age of 67.1 ± 9.7 years (mean ± SD). All patients met the clinically probable PD diagnostic criteria of the International Parkinson and Movement Disorder Society Clinical Diagnostic Criteria for Parkinson’s Disease. Demographic data including age, gender, disease duration, levodopa equivalent daily dosage (LEDD) (12), Hoehn and Yahr Stage (H&Y stage), and history of rhinal diseases were collected from each participant. This study received approval from the Institutional Review Board of Taichung Veterans General Hospital (Approval No.: CE23443C), and all personal information was encrypted to ensure patient privacy.

### 2.2 Clinical assessments

All participants received clinical evaluations when on medication, including the MDS-UPDRS, 39-item Parkinson’s Disease Questionnaire summary index (PDQ-39SI) (13), Non-Motor Symptoms Scale (NMSS) (14), Montreal Cognitive Assessment (MoCA) (15), Parkinson’s Disease Sleep Scale (PDSS) (16), Beck Depression Inventory-II (BDI-II) (17), Beck Anxiety Inventory (BAI) (18), Questionnaire for Impulsive-Compulsive Disorders in Parkinson’s Disease–Rating Scale (QUIP-RS) (19), Neuropsychiatric Inventory (NPI) (20), and traditional Chinese version of the University of Pennsylvania Smell Identification Test (UPSIT-TC) (21).

### 2.3 Olfactory testing and grouping according to the emotional valence of each odor

In the UPSIT-TC, 40 odorants are embedded in “scratch and sniff” labels. During the test, the participants are required, after releasing each odorant using a pencil tip, to smell and identify the correct odor among four choices. During the validation of the UPSIT-TC, 8 odors were replaced in Chinese version of UPSIT due to the limitation of available odorants and unfamiliarity by Taiwanese subjects (21). After a literature review about the emotional valence of each UPSIT-TC odor (22–25), we categorized 15 odors as being pleasant (bubble gum, cherry, banana, fruit punch, licorice, strawberry, chocolate, root beer, pineapple, orange, watermelon, grape, lemon, jasmine, and magnolia), 10 odors as being neutral (menthol, mint, coconut, cedar, lilac, peach, pine, soap, rose, and peanut), 10 odors as being unpleasant (pizza, motor oil, leather, onion, gasoline, turpentine, paint thinner, smoke, natural gas, and rubber tire), and the remaining 5 odors (grapefruit, sandalwood, fish, baby powder, coffee) were not used in the final analysis due to limited literature discussing their emotional valence classification.

The correctness was recorded separately according to the pleasant, neutral, and unpleasant odors. The identification accuracy as percent of correct answers was calculated in the three odor groups regarding emotional valences. The participants were classified as the predominantly pleasant odor impairment group if having the lowest identification accuracy of pleasant odors in the three odor groups. PD patients showing poorest identification accuracy of neutral odors were sorted into the predominantly neutral odor impairment group. Subjects with worst identification accuracy of unpleasant odors belonged to the predominantly unpleasant odor impairment group. If the identification accuracy for any two odor groups were the same, the participants were excluded from the odor impairment grouping. This strategy aimed to screen for the most specific odor emotional valence across different domains of PD symptoms as the initial step of statistical analysis. In the second step, PD symptoms displaying statistically significant differences between odor groups underwent Pearson correlation analysis, followed by linear regression analysis to explore their clinical relevance.

### 2.4 Motor symptom subgrouping

The MDS-UPDRS part III scores were further divided into several domains for group comparisons, correlation, and regression analyses with the olfactory deficits, including tremor (sum of MDS-UPDRS items 3.15–3.18), rigidity (MDS-UPDRS item 3.3), bradykinesia (sum of MDS-UPDRS items 3.2, 3.4–3.9 and 3.14), axial (sum of MDS-UPDRS items 3.1 and 3.9–3.13), tremor dominant (sum of MDS-UPDRS items 2.10 and 3.15–3.18, divided by 11), and akinetic-rigid (sum of MDS-UPDRS items 3.2–3.8 and 3.14, divided by 15) (26, 27).

### 2.5 Statistical analysis

All statistical analyses were performed using SPSS version 22 (IBM Corporation, Armonk, New York, United States). Continuous variables were compared using the Kruskal-Wallis test with Dunn’s post-hoc analysis. The Bonferroni adjustment was done for correction of multiple comparison. Categorical data were analyzed using the chi-square (χ^2^) test. Pearson correlation analysis was performed between the number of misidentified UPSIT-TC odors according to the emotional valence and MDS-UPDRS scores and subscores. Significant results in the correlation analysis were re-analyzed using partial correlation analysis considering age, gender, LEDD, and disease duration as covariates. Linear regression analysis was used to develop models incorporating parameters such as odor identification status, age, gender, LEDD, and disease duration. These models were utilized to predict Parkinson’s disease subscores, which were found to be significant in the partial correlation analysis.

## 3 Results

### 3.1 Demographic data of the participants

A total of 176 PD patients were enrolled in the study. According to the UPSIT-TC test results, 87 patients (55.4%) were classified into the predominantly pleasant odor impairment group, 33 patients (21.0%) were classified into the predominantly neutral odor impairment group, and 37 patients (23.6%) were classified into the predominantly unpleasant odor impairment group. Nineteen patients were excluded from the grouping because they could not be classified into specific groups. The predominantly unpleasant odor impairment group showed a trend of male predominance compared to the other two groups (pleasant odor impairment group vs. neutral odor impairment group vs. unpleasant odor impairment group: 56.32% vs. 54.54% vs. 78.38%, *p* = 0.048). There were no significant group differences in other baseline demographic data, including age, disease duration, LEDD, H&Y stage, and history of rhinal diseases (Table 1).

**Table 1:** Demographic data of the PD patients with different valence of olfactory dysfunction.

	Pleasant Odor Impairment (*N* = 87)	Neutral Odor Impairment (*N* = 33)	Unpleasant Odor Impairment (*N* = 37)	*p* value
Gender, Male (*N*, %)^#,^^	49 (56.32%)	18 (54.54%)	29 (78.38%)	**0.048**
Age, years (Mean ± SD)	66.71 ± 10.18	66.24 ± 8.79	66.49 ± 8.92	0.971
Disease duration, years (Mean ± SD)	4.02 ± 4.38	6.15 ± 8.16	4.73 ± 5.63	0.472
LEDD (mg/day)	473.98 ± 401.65	685.86 ± 494.34	452.66 ± 394.88	0.056
H&Y stage	2.13 ± 0.78	2.36 ± 0.90	2.14 ± 0.54	0.448
MDS-UPDRS, total score (Mean ± SD)	47.06 ± 23.46	59.12 ± 32.07	54.65 ± 27.12	0.100
MDS-UPDRS part I score (Mean ± SD)	14.23 ± 16.80	17.82 ± 20.75	16.84 ± 19.69	0.373
MDS-UPDRS part II score (Mean ± SD)	9.90 ± 7.74	13.70 ± 12.07	12.38 ± 10.11	0.320
MDS-UPDRS part III score (Mean ± SD)^*^	26.79 ± 13.59	35.33 ± 16.36	31.57 ± 12.37	**0.009**
MDS-UPDRS part IV score (Mean ± SD)	0.93 ± 2.57	1.97 ± 3.50	1.54 ± 3.19	0.422
PDQ-39SI (Mean ± SD)	19.78 ± 17.44	26.76 ± 20.93	22.58 ± 18.16	0.239
NMSS (Mean ± SD)	35.18 ± 27.48	44.09 ± 35.374	44.30 ± 34.85	0.129
MOCA (Mean ± SD)	24.64 ± 5.05	22.12 ± 6.23	24.68 ± 4.70	0.950
PDSS (Mean ± SD)	113.41 ± 21.93	108.36 ± 23.13	107.76 ± 25.40	0.388
BDI-II (Mean ± SD)	11.82 ± 10.03	11.97 ± 8.92	12.22 ± 8.75	0.829
BAI (Mean ± SD)	9.72 ± 10.21	9.79 ± 8.02	11.97 ± 9.81	0.277
QUIP-RS (Mean ± SD)	1.47 ± 5.88	1.39 ± 3.35	0.97 ± 2.37	0.620
NPI (Mean ± SD)	5.97 ± 7.33	7.85 ± 7.94	7.00 ± 8.16	0.163
UPSIT-TC (Mean ± SD)	17.33 ± 6.18	15.21 ± 5.84	15.97 ± 6.47	0.156
Rhinal Disease History (N, %)	15 (17.2%)	5 (15.2%)	5 (13.5%)	0.866

N: number of patients; * Predominantly pleasant odor impairment vs predominantly neutral odor impairment, *p* < 0.05; # Predominantly pleasant odor impairment vs predominantly unpleasant odor impairment, *p* < 0.05; ^ Predominantly neutral odor impairment vs predominantly unpleasant odor impairment, *p* < 0.05; PD, Parkinson’s disease; SD, standard deviation; LEDD, levodopa equivalent daily dose; H&Y stage, Hoehn and Yahr stage; MDS-UPDRS, Movement Disorder Society Unified Parkinson’s Disease Rating Scale; PDQ-39SI, 39-item Parkinson’s Disease Questionnaire summary index; NMSS, Non-Motor Symptoms Scale; MoCA, Montreal Cognitive Assessment; PDSS, Parkinson’s Disease Sleep Scale; BDI-II, Beck Depression Inventory-II; BAI, Beck Anxiety Inventory; QUIP-RS, Questionnaire for Impulsive-Compulsive Disorders in Parkinson’s Disease–Rating Scale; NPI, Neuropsychiatric Inventory; UPSIT-TC, traditional Chinese version of the University of Pennsylvania Smell Identification Test.The bold values indicate the *p*-value < 0.05.

### 3.2 Motor severity was different regarding emotional valence of olfactory impairment

Group comparisons revealed no significant differences in MDS-UPDRS total, part II, IV, PDQ-39SI and most non-motor evaluations including NMSS, MoCA, PDSS, BDI-II, BAI, QUIP-RS, NPI, and UPSIT-TC. There was a trend of higher MDS-UPDRS part III scores in the predominantly neutral and unpleasant odor impairment groups than in the predominantly pleasant odor impairment group (pleasant odor impairment group vs. neutral odor impairment group vs. unpleasant odor impairment group: 26.79 ± 13.59 vs. 35.33 ± 16.36 vs. 31.57 ± 12.37, *p* = 0.009). The differences remained significant between the predominantly pleasant and neutral odor impairment groups in the post-hoc analysis (Table 1).

Differences in motor subdomains including tremor, rigidity, bradykinesia, axial, tremor dominant, and akinetic-rigid severity were further compared among the three groups. Trends of higher subscores of rigidity (*p* = 0.003), bradykinesia (*p* = 0.012), and akinetic-rigid (*p* = 0.001) symptoms were noted in the predominantly neutral and unpleasant odor impairment groups compared to the predominantly pleasant odor impairment group (Table 2). In the post-hoc analysis, the difference in rigidity subscore remained significant between the predominantly pleasant and unpleasant odor impairment groups, while significant differences were found in the bradykinesia and akinetic-rigid symptom scores between the predominantly pleasant and neutral odor impairment groups (Table 2).

**Table 2:** MDS-UPDRS motor subscores of the PD patients with different valence of olfactory deficits.

MDS-UPDRS motor subscore	Pleasant odor impairment (*N* = 87)	Neutral odor impairment (*N* = 33)	Unpleasant odor impairment (*N* = 37)	*p* value
Tremor (Mean ± SD)	3.52 ± 3.62	3.55 ± 3.87	3.78 ± 3.76	0.889
Rigidity (Mean ± SD)^#^	6.03 ± 3.76	7.94 ± 4.28	8.49 ± 3.81	**0.003**
Bradykinesia (Mean ± SD)^*^	14.17 ± 7.47	19.39 ± 9.35	15.95 ± 7.30	**0.012**
Axial (Mean ± SD)	3.41 ± 3.60	5.18 ± 5.08	3.84 ± 3.40	0.201
Tremor dominant (Mean ± SD)	0.41 ± 0.38	0.44 ± 0.40	0.46 ± 0.39	0.707
Akinetic-rigid (Mean ± SD)^*^	1.19 ± 0.56	1.61 ± 0.62	1.47 ± 0.51	**0.001**

N: number of patients; * Predominantly pleasant odor impairment vs predominantly neutral odor impairment, *p* < 0.05; # Predominantly pleasant odor impairment vs predominantly unpleasant odor impairment, *p* < 0.05; PD: Parkinson’s disease; SD: standard deviation; MDS-UPDRS: Movement Disorder Society Unified Parkinson’s Disease Rating Scale; Tremor subscore = Sum of MDS-UPDRS 3.15–3.18; Rigidity subscore = Sum of MDS-UPDRS 3.3; Bradykinesia subscore = Sum of MDS-UPDRS 3.2, 3.4–3.9, and 3.14; Axial subscore = Sum of MDS-UPDRS 3.1 and 3.9–3.13; Tremor dominant subscore = Sum of MDS-UPDRS 2.10, and 3.15–3.18, divided by 11; Akinetic-rigid subscore = Sum of MDS-UPDRS 3.2–3.8 and 3.14, divided by 15.The bold values indicate the *p*-value < 0.05.

### 3.3 Severity of neutral and unpleasant odor impairment was more strongly correlated with PD motor and non-motor problems than pleasant odor impairment

Finally, correlation analysis was performed between the number of misidentified UPSIT-TC odors according to the emotional valence and MDS-UPDRS scores and subscores. Correlations were found between MDS-UPDRS part II, III, total scores and the number of misidentified neutral and unpleasant odors (MDS-UPDRS part II score vs. number of misidentified neutral odors: *R* = 0.234, *p* = 0.002; MDS-UPDRS part II score vs. number of misidentified unpleasant odors: *R* = 0.239, *p* = 0.001; MDS-UPDRS part III score vs. number of misidentified neutral odors: *R* = 0.226, *p* = 0.003; MDS-UPDRS part III score vs. number of misidentified unpleasant odors: *R* = 0.212, *p* = 0.005; MDS-UPDRS total score vs. number of misidentified neutral odors: R = 0.226, *p* = 0.003; MDS-UPDRS total score vs. number of misidentified unpleasant odors: *R* = 0.235, *p* = 0.002) (Table 3). The significance remained after adjusting for age and gender as covariates, but did not persist with age, gender, LEDD, and disease duration as covariates in partial correlation and linear regression analyses (Tables 3, 4).

**Table 3:** Correlations between MDS-UPDRS motor subscore and the emotional valence of olfactory impairment (N = 176).

MDS-UPDRS Subscore	Pleasant odor misidentification		Neutral odor misidentification		Unpleasant odor misidentification	
	Pearson’s r	*p* value	Pearson’s r	*p* value	Pearson’s r	*p* value
Part I	0.05	0.512	0.141	0.062	0.077	0.307
Part II	0.148	**0.05**	0.234	**0.002** ^*^	0.239	**0.001** ^*^
Part III	0.192	**0.011**	0.226	**0.003** ^*^	0.212	**0.005** ^*^
Part IV	−0.007	0.93	0.115	0.13	0.072	0.344
Total	0.158	**0.036**	0.226	**0.003** ^*^	0.235	**0.002** ^*^
Tremor	0.142	0.06	0.086	0.259	0.102	0.178
Rigidity	0.19	**0.011**	0.286	**<0.001** ^*#^	0.338	**<0.001** ^*#^
Bradykinesia	0.118	0.118	0.154	**0.041**	0.107	0.158
Axial	0.139	0.065	0.146	0.053	0.117	0.123
Tremor dominant	0.133	0.078	0.133	0.078	0.114	0.131
Akinetic-rigid	0.154	**0.041**	0.236	**0.002** ^*#^	0.227	**0.002** ^*#^

N: number of patients; * Adjusting for age and gender as covariates, *p* < 0.05; # Adjusting for age, gender, LEDD, and disease duration as covariates, *p* < 0.05; MDS-UPDRS: Movement Disorder Society Unified Parkinson’s Disease Rating Scale; Tremor subscore = Sum of MDS-UPDRS 3.15–3.18; Rigidity subscore = Sum of MDS-UPDRS 3.3; Bradykinesia subscore = Sum of MDS-UPDRS 3.2, 3.4–3.9, and 3.14; Axial subscore = Sum of MDS-UPDRS 3.1 and 3.9–3.13; Tremor dominant subscore = Sum of MDS-UPDRS 2.10, and 3.15–3.18, divided by 11; Akinetic-rigid subscore = Sum of MDS-UPDRS 3.2–3.8 and 3.14, divided by 15.The bold values indicate the *p*-value < 0.05.

**Table 4:** Linear regression models for clinical variables predicting disease severity subscore in PD patients (N = 176).

	MDS-UPDRS part II		MDS-UPDRS part III		MDS-UPDRS total		Rigidity		Akinetic-rigid	
	B	*p* value	B	*p* value	B	*p* value	B	*p* value	B	*p* value
Model 1										
Constant	−8.367	0.085	2.030	0.784	−0.520	0.970	1.064	0.603	0.497	0.108
Misidentified odors^*^	0.808	**0.010**	1.179	**0.015**	2.280	**0.011**	0.457	**0.001**	0.055	**0.006**
Gender, F/M	−0.330	0.811	1.783	0.400	−3.270	0.406	1.244	**0.034**	0.095	0.283
Age	0.230	**0.001**	0.295	**0.007**	0.617	**0.002**	0.040	0.173	0.007	0.115
Model 2										
Constant	−7.581	0.115	3.442	0.641	1.531	0.911	1.406	0.483	0.559	0.070
Misidentified odors^#^	0.791	**0.010**	0.986	**0.036**	2.334	**0.007**	0.511	**<0.001**	0.048	**0.014**
Gender, F/M	−0.709	0.613	1.378	0.524	−4.433	0.267	0.973	0.098	0.074	0.410
Age	0.223	**0.002**	0.293	**0.008**	0.591	**0.004**	0.033	0.262	0.007	0.129
Model 3										
Constant	−14.953	**0.001**	−1.105	0.884	−14.826	0.267	1.117	0.595	0.378	0.230
Misidentified odors^*^	0.356	0.220	0.927	0.062	1.179	0.176	0.447	**0.001**	0.045	**0.029**
Gender, F/M	0.653	0.602	2.407	0.260	−0.639	0.865	1.295	**0.030**	0.120	0.178
Age	0.285	**<0.001**	0.321	**0.003**	0.738	**<0.001**	0.040	0.179	0.008	0.073
Disease duration	0.301	**0.020**	−0.038	0.864	0.081	0.834	−0.070	0.248	−0.003	0.714
LEDD	0.007	**<0.001**	0.005	0.088	0.020	**<0.001**	0.001	0.450	0.000	0.088
Model 4										
Constant	−14.743	**0.001**	−0.332	0.965	−14.243	0.282	1.393	0.500	0.414	0.188
Misidentified odors^#^	0.424	0.126	0.802	0.091	1.550	0.062	0.514	**<0.001**	0.041	**0.037**
Gender, F/M	0.431	0.734	2.128	0.328	−1.517	0.689	1.035	0.082	0.104	0.249
Age	0.278	**<0.001**	0.320	**0.004**	0.706	**<0.001**	0.032	0.275	0.008	0.082
Disease duration	0.286	**0.027**	−0.070	0.748	0.026	0.947	−0.089	0.137	−0.005	0.585
LEDD	0.007	**<0.001**	0.006	**0.049**	0.020	**<0.001**	0.001	0.289	0.000	**0.047**

N: number of patients; The *p*-value was significant (<0.05) in all equations utilizing models 1 through 4. * Number of misidentified neutral odors; # Number of misidentified unpleasant odors; PD: Parkinson’s disease; MDS-UPDRS: Movement Disorder Society Unified Parkinson’s Disease Rating Scale; F/M: Female/Male; LEDD: levodopa equivalent daily dose; Rigidity subscore = Sum of MDS-UPDRS 3.3; Akinetic-rigid subscore = Sum of MDS-UPDRS 3.2–3.8 and 3.14, divided by 15.The bold values indicate the *p*-value < 0.05.

Although the significant correlations between MDS-UPDRS II, III, total scores and number of misidentified odors according to the UPSIT-TC emotional valence did not persist using age, gender, LEDD, and disease duration as covariates, significant correlations were found when using these covariates between the number of misidentified UPSIT-TC odors and subscores of MDS-UPDRS part III, including the akinetic-rigid and rigidity subscores, and neutral and unpleasant odor identification errors. (MDS-UPDRS akinetic-rigid subscore vs. number of misidentified neutral/unpleasant odor: *R* = 0.236, *p* = 0.002/ *R* = 0.227, *p* = 0.002; MDS-UPDRS rigidity subscore vs. number of misidentified neutral/unpleasant odor: *R* = 0.286, *p* < 0.001/ *R* = 0.338, *p* < 0.001) (Table 3). The association remained significant in the linear regression models, which included odor identification status, age, gender, disease duration, and LEDD as independent variables (Table 4).

Not limited to the motor symptoms, significant correlation existed between misidentified UPSIT-TC neutral odor number vs. MoCA score (*R* = −0.235, *p* = 0.002), wrongly identified unpleasant odor number vs. MDS-UPDRS item 1.1 cognitive impairment score (*R* = 0.212, *p* = 0.005), and item 1.11 constipation problem score vs. number of impaired neutral/unpleasant odor (*R* = 0.234, *p* = 0.002/*R* = 0.202, *p* = 0.007). The significance persisted after adjusting for age, gender, LEDD, and disease duration as covariates in both partial correlation and linear regression analyses (Supplementary Table S1).

## 4 Discussion

In the current study, 55.4% of the PD patients showed greater olfactory impairment in pleasant odor identification. However, compared to the predominantly pleasant odor impairment group, the predominantly neutral/unpleasant odor impairment groups showed trends of worse motor symptoms, including MDS-UPDRS part III score, rigidity, bradykinesia, and akinetic-rigid subscores. The misidentification rates of neutral and unpleasant odors were more strongly correlated with PD motor and non-motor symptoms.

Hyposmia occurs in up to 96% of PD patients (1). While it is widely accepted that hyposmia is an important PD prodromal symptom, the link between olfactory impairment and motor/non-motor symptoms in PD is still controversial (5–8). The posterior putamen has been shown to be involved in odor emotional valence processing and motor reserve in PD (28, 29). Olfactory senescence intensifies with age, particularly affecting the perception of pleasant odors (10), and a similar phenomenon has also been reported in PD (11). This is consistent with our data, as more patients were classified into the predominantly pleasant odor impairment group (55.4%, *N* = 87). While unpleasant odors are considered to be more resistant to aging than pleasant odors (30), impairment of unpleasant odors may reflect more severe and diverse neurodegeneration, as shown in our correlation analysis. Previous studies discussing the relationship of olfactory impairment and dysfunction of different motor subtypes in PD showed mixing results. As previously reported in a congress poster, there was an association between impaired odor identification and rigidity among all cardinal motor impairments of PD (31). While one study mentioned the postural instability and gait disorders patients had the poorer olfactory identification than the tremor dominant (TD) patients if not considering gender (5), another research disclosed olfactory dysfunction correlated with motor decline only in the TD subtype (6). Unpleasant smells may be related to olfactory hallucination. Unpleasant smells may be related to olfactory hallucination. The frequency of olfactory hallucinations (OH) in PD patients has been reported to range from 0.5 to 18.2% in various studies (32). Solla et al. reported that the majority of OH instances (81.3%) were pleasant, and PD patients experiencing OH had higher UPDRS III scores compared to those without OH (33). Given the lower neutral/unpleasant odor identification accuracy observed in our study, which correlated with motor severities, we hypothesized that the misidentification of neutral or unpleasant odors as pleasant smells might be indicative of olfactory hallucination. Consequently, this could be associated with both motor and non-motor symptoms as reported in previous research (5, 7). By using a more specific category of valence odors related to severity of degeneration, it may be possible to detect the relationship between olfactory dysfunction and different clusters of motor symptoms of PD.

In one olfactory functional magnetic resonance imaging (fMRI) study evaluating Alzheimer’s disease (AD) patients, the number of activated voxels in the right inferior frontal gyrus, orbital part (F3O), was greater with unpleasant odors than with pleasant odors in the normal control (NC) group, however this pattern was not seen in mild cognitive impairment (MCI) and AD patients. The authors further reported that primary olfactory cortex activation with medium concentrations of unpleasant odors could differentiate the NC group from the MCI group, and the MCI group from the AD group. Moreover, correlations between right F3O activation and Mini Mental State Examination and MoCA scores were higher with unpleasant odor stimulation than with pleasant odor stimulation (34). While previous studies have shown that hyposmia is an important clinical marker of cognitive decline in PD (7), our results further disclosed stronger correlations between cognitive problems with neutral/unpleasant odor impairment than with pleasant odor impairment. In the non-motor domain analysis, a positive correlation was also observed between the severity of constipation and degree of neutral/unpleasant odor impairments. A correlation between olfactory impairment and the severity of constipation was also reported in one study focusing on PD patients receiving subthalamic nucleus deep brain stimulation (STN-DBS) (35). In that study, the authors proposed that a lower abundance and variety of microbiota could be the cause. Since STN-DBS has been reported to be able to improve olfactory dysfunction and the effects would be minimized in severe constipation patients, we hypothesize that olfactory dysfunction and constipation may be mutually interacting symptoms, implying the severity of gut-brain axis damage in PD patients. Our results and the aforementioned AD-fMRI study highlight the plausibility of an association between neutral/unpleasant odor impairments and clinical severity scoring systems of neurodegenerative diseases.

There are several limitations to the current study. First, relatively few studies have discussed the emotional valence of individual odors in the UPSIT-TC compared to the English version, and therefore five odors were excluded from our analysis. Consequently, only 35 of the 40 items in the UPSIT-TC, comprising 15 pleasant odors, 10 neutral odors, and 10 unpleasant odors, were entered into the final analysis. Currently, there is no paper discussing the classification of the PD patients with different valence of olfactory deficits, rendering our classification arbitrary and lacking delicacy. Given the unequal number in each odor group, identification accuracy was used in the group comparison analysis. In odor impairment grouping, 19 patients were excluded due to equal identification accuracy in two emotional valence odor groups. While our study observed a predominance of males in the group with predominantly unpleasant odor impairment, potentially introducing heterogeneity into data analysis. A previous study suggested no sex difference in odor identification (30), and, to address this, we included gender as a covariate in the correlation analysis. Second, given the retrospective nature of this study and as all data were collected during clinical visits, all patients were evaluated when on medication, and the number of patients in the three odor impairment groups was not homogeneous. However, a previous study reported that olfactory tests were not affected by PD medications (36). In the current study, there was no significant difference in LEDD among the three groups, and it was used as one of the covariates in the partial correlation and regression analysis. A non-parametric statistical method was applied for group comparisons. Another limitation of our clinical visit-based data is that we could not refer all patients for comprehensive rhinal examinations. We traced all available medical records, and the number of patients with rhinal diseases did not significantly differ among the three groups, and the significance of the correlation results remained after using a history of rhinal diseases as a covariate. Finally, this was a retrospective, single-center study with a limited number of patients. In addition, the clinical visit-based data limited the possibility of obtaining information from the participants when off medication, and we lacked adequate funding for recruiting healthy volunteers for similar clinical evaluations. The aim of this study was to provide pilot information which could be applicable in daily clinical practice regarding the possibility of using simplified olfactory testing focusing on specific emotional valence odors to assist in evaluating the risk of motor disability in PD patients. Future large-scale, multi-center studies recruiting both healthy volunteers and more patients are warranted to evaluate both on and off medication status and verify our findings.

## 5 Conclusion

In conclusion, our results showed an association between olfactory impairment of specific emotional valence and PD severity in different subdomains, especially akinetic-rigid symptoms. A concise olfactory test focusing on both neutral and unpleasant odors may provide greater insights into PD symptoms.

## Data availability statement

The raw data supporting the conclusions of this article will be made available by the authors, without undue reservation.

## Ethics statement

The studies involving humans were approved by the Institutional Review Board of Taichung Veterans General Hospital. The studies were conducted in accordance with the local legislation and institutional requirements. Written informed consent for participation was not required from the participants or the participants' legal guardians/next of kin in accordance with the national legislation and institutional requirements.

## Author contributions

S-CC: Formal analysis, Writing – original draft. T-CF: Formal analysis, Writing – original draft. H-BL: Writing – original draft. Y-HL: Writing – original draft. M-HC: Writing – original draft. Y-JG: Conceptualization, Formal analysis, Writing – review & editing, Methodology, Writing – original draft.
