# Supplementary material for: The greatest loss of unpleasant smells may be related to the risk of more severe PD symptoms
Source: Front Neurol. 2024 Apr 2;15:1362763. doi: 10.3389/fneur.2024.1362763 (PMC11018954; doi:10.3389/fneur.2024.1362763)
Supplement: Supplementary file 1 [file Table_1.docx]

**Supplementary table 1.** Linear regression models for clinical variables predicting cognition and constipation in PD patients (N=176)

|  | MoCA | | MDS-UPDRS item 1.1 | | MDS-UPDRS item 1.11 | |
| --- | --- | --- | --- | --- | --- | --- |
|  | B | p value | B | p value | B | p value |
| Model 1 | | | | | | |
| Constant | 42.401 | **<0.001** | -0.202 | 0.626 | 0.610 | 0.295 |
| Misidentified odors^*^ | -0.393 | **0.011** | 0.040 | 0.135 | 0.117 | **0.002** |
| Gender, F/M | 2.225 | **0.001** | -0.145 | 0.219 | -0.021 | 0.900 |
| Age | -0.260 | **<0.001** | 0.016 | **0.007** | -0.002 | 0.834 |
| Model 2 | | | | | | |
| Constant | 41.841 | **<0.001** | -0.205 | 0.612 | 0.749 | 0.198 |
| Misidentified odors^#^ | -0.272 | 0.071 | 0.066 | **0.011** | 0.099 | **0.007** |
| Gender, F/M | 2.310 | **0.001** | -0.188 | 0.113 | -0.062 | 0.714 |
| Age | -0.263 | **<0.001** | 0.014 | **0.016** | -0.002 | 0.816 |
| Model 3 | | | | | | |
| Constant | 42.973 | **<0.001** | -0.243 | 0.566 | 0.282 | 0.632 |
| Misidentified odors^*^ | -0.333 | **0.036** | 0.040 | 0.146 | 0.090 | **0.020** |
| Gender, F/M | 2.053 | **0.003** | -0.153 | 0.202 | 0.049 | 0.768 |
| Age | -0.265 | **<0.001** | 0.016 | **0.007** | 0.001 | 0.898 |
| Disease duration | 0.074 | 0.291 | 0.017 | 0.155 | -0.009 | 0.604 |
| LEDD | -0.002 | 0.094 | 0.000 | 0.500 | 0.001 | **0.012** |
| Model 4 | | | | | | |
| Constant | 42.655 | **<0.001** | -0.232 | 0.580 | 0.355 | 0.546 |
| Misidentified odors^#^ | -0.235 | 0.124 | 0.064 | **0.015** | 0.080 | **0.030** |
| Gender, F/M | 2.101 | **0.003** | -0.193 | 0.108 | 0.019 | 0.909 |
| Age | -0.268 | **<0.001** | 0.015 | **0.015** | 0.001 | 0.927 |
| Disease duration | 0.085 | 0.232 | 0.015 | 0.208 | -0.012 | 0.479 |
| LEDD | -0.002 | **0.046** | 0.000 | 0.509 | 0.001 | **0.005** |

MoCA: Montreal Cognitive Assessment; MDS-UPDRS item 1.1 cognitive impairment subscore; MDS-UPDRS item 1.11 constipation problem subscore; N: number of patients; The p-value was significant (<0.05) in all equations utilizing models 1 through 4. ^*^ Number of misidentified neutral odors; ^#^ Number of misidentified unpleasant odors; PD: Parkinson's disease; MDS-UPDRS: Movement Disorder Society Unified Parkinson's Disease Rating Scale; F/M: Female/Male; LEDD: levodopa equivalent daily dose.
